# Novel Agents for Acute Myeloid Leukemia

**DOI:** 10.3390/cancers10110429

**Published:** 2018-11-09

**Authors:** Mario Luppi, Francesco Fabbiano, Giuseppe Visani, Giovanni Martinelli, Adriano Venditti

**Affiliations:** 1 Section of Hematology Department of Medical and Surgical Sciences, Azienda Ospedaliera Universitaria di Modena Università di Modena e Reggio Emilia, 41125 Modena, Italy; mario.luppi@unimore.it; 2 Division of Hematology and Bone Marrow Transplantation, Ospedali Riuniti Villa Sofia-Cervello, 90146 Palermo, Italy; francesco.fabbiano@ospedaliriunitipalermo.it; 3 Hematology and Transplant Center, Azienda Ospedaliera Marche Nord, 61121 Pesaro, Italy; giuseppe.visani@ospedalimarchenord.it; 4 Istituto Scientifico Romagnolo per lo Studio e la Cura dei Tumori (IRST) IRCCS, 47014 Meldola (FC), Italy; giovanni.martinelli@irst.emr.it; 5 Hematology, Department of Biomedicine and Prevention, “Tor Vergata” University of Rome, 00133 Roma, Italy

**Keywords:** acute myeloid leukemia, “7 + 3” regimen, gemtuzumab ozogamicin, CPX-351, FLT3, midostaurin, enasidenib, palliative care

## Abstract

Acute myeloid leukemia (AML) is a complex hematological disease characterized by genetic and clinical heterogeneity. Recent advances in the understanding of AML pathogenesis have paved the way for the development of new agents targeting specific molecules or mechanisms that contribute to finally move beyond the current standard of care, which is “3 + 7” regimen. In particular, new therapeutic options such as targeted therapies (midostaurin and enasidenib), monoclonal antibodies (gemtuzumab ozogamicin), and a novel liposomal formulation of cytarabine and daunorubicin (CPX-351) have been recently approved, and will be soon available for the treatment of adult patients with AML. In this review, we will present and describe these recently approved drugs as well as selected novel agents against AML that are currently under investigation, and show the most promising results as monotherapy or in combination with chemotherapy. The selection of these emerging treatments is based on the authors’ opinion.

## 1. Introduction

Acute myeloid leukemia (AML) is a malignant disorder characterized by an impaired differentiation of hematopoietic stem cells, leading to an abnormal accumulation of immature malignant cells and the reduced production of healthy mature blood cells [1]. 

Among leukemias, AML is one of the most common diseases, accounting for approximately 30% of leukemia adult cases [2]. AML is primarily a disease of later adulthood: the incidence rate by age increases from 50 years, reaching the peak at around 80 years of age, and the median age at diagnosis is 65 years [3,4]. With an incidence rate that has increased over the past two decades by 30% [5], AML represents a substantial health problem that requires strict monitoring and innovative treatment strategies.

Despite a number of important advances in the understanding of AML pathogenesis, the frontline therapeutic strategy has not substantially changed in 40 years. An initial intensive induction with seven-day infusion of cytarabine plus three days of an anthracycline treatment (commonly referred to as “7 + 3” regimen) is commonly used to produce complete remission (CR) and represents the backbone of upfront AML treatment [6]. A CR is usually achieved in 60–80% of younger adults and in 40–60% of AML patients older than 65 years [6]. Postremission regimens include conventional chemotherapy (consolidation therapy) and, where feasible, conditioning therapy followed by allogeneic hematopoietic stem cell transplantation (HSCT). However, several disease-specific and patient-specific factors such as cytogenetic/molecular features (European LeukemiaNet, ELN genetic risk), age, performance status, and comorbidities often limit the use of high-dose cytotoxic chemotherapy [6]. Increasing age is frequently associated with comorbidities, adverse biological features, and multidrug resistance that negatively affect clinical outcomes; consequently, the five-year survival rate in intensively-treated AML patients older than 65 years is only 10% [1]. 

The identification of new driver mutations through next-generation sequencing has shed light on the pathogenesis of AML, revealing a considerable genomic complexity and genetic heterogeneity, and leading to the development of novel potential targeted therapies against AML. It is worthy of note that targeted therapies rarely act on a single specific pathway; usually, they affect multiple signaling pathways [7]. Recently, the first targeted therapy in AML, midostaurin, has been approved for the treatment of AML by the Food and Drug Administration (FDA) and by the European Medicines Agency (EMA), and will be soon available in the European countries. The antibody drug conjugate gemtuzumab ozogamicin also has been recently re-approved by the FDA and EMA [8]. Other innovative strategies such as an inhibitor of mutant proteins (enasidenib) and novel formulations of traditional chemotherapy (the liposome-based CPX-351, which is a combination of cytarabine and daunorubicin) received FDA approval in 2017. Over the last year, the therapeutic armamentarium in AML is dramatically expanding. In addition to describing the latest approved agents against AML, in this review, we will select and focus on those new agents that, in our opinion, are the most promising future treatments for AML. From our point of view, the panel of agents in development that are presented in this review are likely going to pave the way toward new treatment paradigms in AML. Although there have also been advancements of the therapeutic strategies in acute promyelocytic leukemia, this topic will not be discussed in this review.

## 2. Monoclonal Antibodies

Monoclonal antibodies specifically target antigens expressed on the surface of leukemic cells; they promote anti-neoplastic activity by immunomodulating the tumor microenvironment, by exerting cell-mediated cytotoxicity, or by delivering conjugated potent chemotherapy to tumor cells. In recent years, a variety of antigen-specific immunotherapies, including antibodies against leukemic myeloblast antigens (CD33, CD123) and the leukemia stem cell markers (CD123, CD25, CD44, CD96, CD47, CD32) have been developed and tested in preclinical studies [9,10]. However, these first generation antibodies that were generated to directly target leukemic cells showed limited anti-tumor activities. A phase II/III study of the anti-CD123 antibody talacotuzumab (JNJ-56022473), in association with decitabine, in patients with AML who are not candidates for intensive chemotherapy, has been recently completed, and results will be available soon (NCT02472145).

Among monoclonal antibodies, there is increasing interest in novel antibody-drug conjugates (ADC). Vadastuximab talirine (SGN-CD33A) is an engineered CD33-directed antibody conjugated to pyrrolobenzodiazepine dimers that showed activity and a tolerable safety profile alone or in combination with standard chemotherapy or hypomethylating agents (HMAs), in AML patients enrolled in phase I studies [11,12,13,14]. The promising results have led to the phase III CASCADE trial investigating vadastuximab talirine in combination with HMAs (azacitidine or decitabine) compared with hypomethylating agents alone, in older patients with newly diagnosed AML (NCT02785900). In June 2017, the CASCADE trial was discontinued for a higher rate of deaths, including fatal infections, in the vadastuximab talirine-treated group versus the control group. Cases of hepatotoxicity, including sinusoidal obstruction syndrome (SOS), have been also associated with vadastuximab talirine treatment [15]. Altogether, the unfavorable safety profile of vadastuximab talirine has raised numerous concerns and slowed down its clinical development.

Among ADCs, gemtuzumab ozogamicin is the only therapy approved by the FDA and EMA for the treatment of newly diagnosed or refractory/relapsed, CD33-positive patients with AML, in combination with daunorubicin and cytarabine or as a stand-alone treatment. Gemtuzumab ozogamicin is a recombinant humanized anti-CD33 antibody conjugated to calicheamicin, which is a potent cytotoxic antibiotic. On the basis of the encouraging results of three single-arm phase I studies, this ADC received an accelerated approval by the FDA in 2000 for relapsed AML patients [16]. However, the phase III study that requested full regulatory approval (Southwest Oncology Group, SWOG, S0106), investigating the effect of gemtuzumab ozogamicin plus conventional induction therapy versus conventional induction therapy alone, in newly diagnosed AML patients (<60 years of age), failed to show an improved response and survival, and demonstrated a significantly higher risk of fatal adverse events [17]. The lack of clear benefits, as well as the emerging safety concerns raised by the interim analysis of the SWOG S0106 study, led to an early termination of this trial and the withdrawal of gemtuzumab ozogamicin from the market in 2010. Over the past eight years, several investigator-led clinical trials, including ALFA-0701 [18], AML-19 [19], and MyloFrance-1 [20] contributed to the favorable reassessment of gemtuzumab ozogamicin, leading to the FDA and EMA recent approval.

So far, clinical studies on immune checkpoint inhibitors (anti-PD-1, anti-PD-L1, anti-CTL4) have provided limited results in AML. However, the immune checkpoint inhibitors given in association with HMAs could represent an interesting strategy to potentiate the activity of immunotherapies [10]. Preliminary results from an ongoing phase I/II study on 53 relapsed/refractory AML patients treated with nivolumab plus 5-azacytidine revealed an overall response rate (ORR) of 35%, including a CR of 21%, and a median overall survival of 5.7 months; all of the patients experiencing severe immune toxicities (grade 3/4) responded rapidly to steroid treatment [21].

## 3. New Formulation of Old Drugs

The traditional “7 + 3” regimen has been the mainstay of AML treatment for years. In order to continue improving outcomes, new formulations of this cytotoxic combination have been recently developed. 

**Figure 1:**
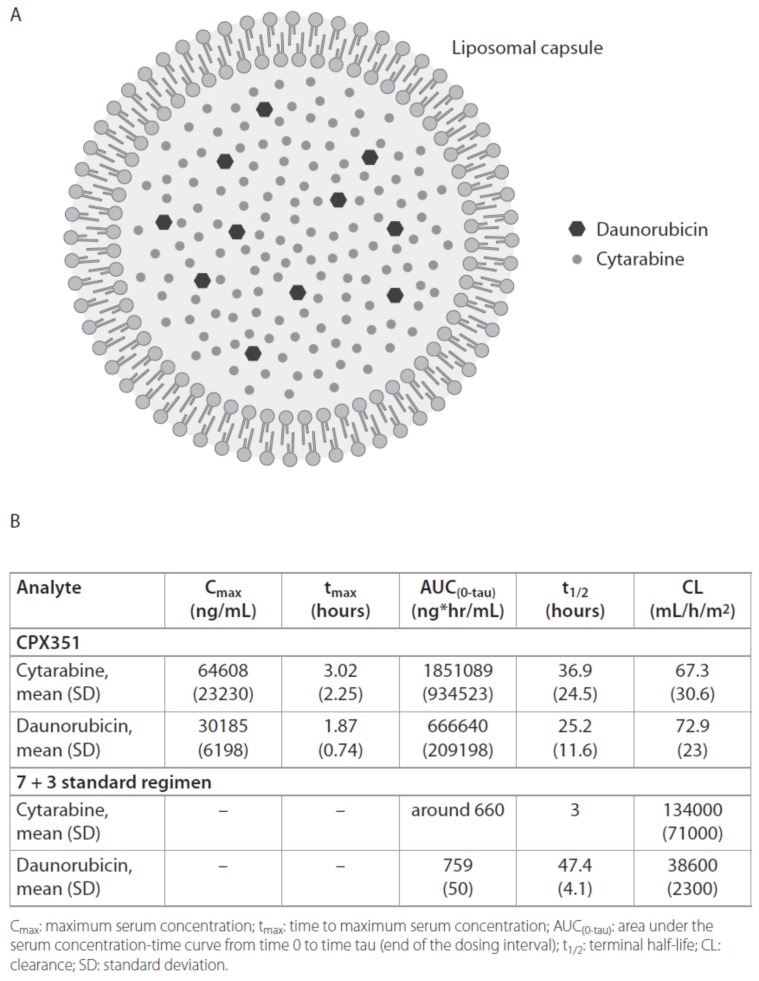
Schematic representation and pharmacokinetics of CPX-351. (**A**) CPX-351 is a liposomal formulation of the cytotoxic drugs cytarabine and daunorubicin at a molar ratio of 1:5; (**B**) A comparison between the pharmacokinetic features of liposomal cytarabine and daunorubicin (on day 5 after infusion of CPX-351 (101 units/m^2^) [24]) versus non-liposomal forms [25,26,27].

**Figure 2:**
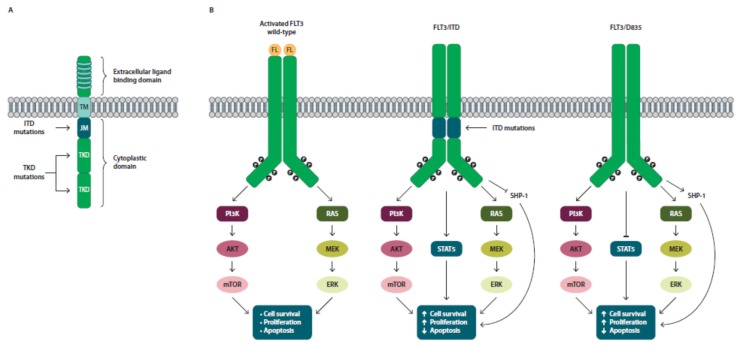
Structure and signaling pathways of activated and mutated FMS-like tyrosine kinase 3 (FLT3). (**A**) Schematic representation of inactivated FLT3; (**B**) Ligand binding to the wild-type FLT3 receptor activates several signaling pathways, such as PI3K/Akt/mTOR and RAS/ mitogen-activated protein kinase kinase (MEK)/extracellular-signal-regulated kinase (ERK), pathways, which are involved in cell survival, proliferation, and apoptosis; FLT3/ITD and FLT3/TKD constitutively stimulate RAS and PI3K downstream pathways, but FLT3/ITD activates STAT5 signaling and reduces the expression of SHP-1, while the mutant form FLT3-D835 activates SHP-1 phosphatase and negatively regulates STAT5 signaling. P: phosphorylation site; TM: transmembrane domain; JM: juxtamembrane domain; TKD: tyrosine kinase domain; ITD: internal tandem duplications; FL: FLT3 ligand.

CPX-351 is a new liposomal formulation that encapsulates cytarabine and daunorubicin in a fixed 5:1 molar ratio (Figure 1A), and in vivo showed superior antileukemic activity compared to free-drug cocktails [22,23]. The encapsulation of cytarabine and daunorubicin allows to maintain the 5:1 molar ratio for up to 24 h [24]. In Figure 2A, the pharmacokinetic features of the two drugs after five days from CPX-351 infusion are shown and compared to those of the two non-liposomal drugs. Among these characteristics, the clearance rates of the encapsulated drugs are remarkably lower (cytarabine: 67.3 mL/h/m^2^; daunorubicin: 72.9 mL/h/m^2^) than those of the unencapsulated forms (cytarabine: 134,000 mL/h/m^2^; daunorubicin: 38,600 mL/h/m^2^) (Figure 2A) [24,25,26,27]. Several phase II studies investigated the safety and the activity of CPX-351 in specific populations of AML patients [28,29,30]. In particular, two multicenter, randomized, open-label, phase II studies simultaneously assessed the effects of CPX-351 in comparison with intensive salvage therapy or conventional “7 + 3” regimen in, respectively, adults with relapsed AML [29] and newly diagnosed older AML patients [28]. Both trials showed a trend toward higher response rates in the CPX-351 groups compared to control groups, but no differences in term of survival. In particular, a remarkable efficacy was detected in subgroups of patients with high risk/secondary AML. Observations from these trials provided a rationale for a phase III study. The pivotal phase III study recruited 309 older patients (60–75 years) with untreated secondary AML (defined as AML with a history of prior cytotoxic treatment, antecedent myelodysplastic syndrome (MDS), or chronic myelomonocytic leukemia (CMML)) or AML with MDS-related cytogenetic abnormalities, as indicated by the World Health Organization (WHO) criteria [31]. CPX-351 (100 units/m^2^ on days 1, 3, and 5) treatment was compared with standard cytarabine and daunorubicin regimen, showing superior overall survival, OS (9.6 versus 5.9 months, hazard ratio (HR) = 0.69; *p* = 0.003) and composite CR, CRc (47.7% versus 33.3%; *p* = 0.016). The 60-day mortality observed in this study favored the CPX-351-treated patients (13.7% versus 21.2%) and grade 3–5 adverse events (AEs) were similar in frequency and severity in both groups [31]. Based on this evidence, CPX-351 recently received FDA and EMA approval for the treatment of adult patients with newly diagnosed therapy-related AML (t-AML) or AML with myelodysplasia-related changes. Considering the activity showed in the phase I study by a lower dose of CPX-351, an ongoing phase II trial is evaluating the outcomes of this liposomal formulation at doses of 50 units/m^2^ and 75 units/m^2^ in newly diagnosed AML patients who are otherwise at a high risk of induction mortality [30].

## 4. FLT3 Inhibitors

Mutations in the FMS-like tyrosine kinase 3 (FLT3), which is a receptor tyrosine kinase involved in the proliferation, differentiation, and apoptosis of hematopoietic cells (Figure 2) [32], are the most frequent genetic lesions in AML, occurring in around one-third of patients [33]. Two different types of FLT3 mutations have been detected in malignant cells from AML patients: internal tandem duplication (ITD) in the juxtamembrane domain, which accounts for approximately 23% of AML cases, and point mutations (typically at codons D835 and I836) in the intracellular tyrosine kinase domain (TKD) observed in 7% of patients (Figure 2A) [6,33,34]. Both mutations cause a ligand-independent activation of the receptor, leading to a constitutive stimulation of the downstream signaling pathway [35,36]. As wild-type FLT3, the mutant forms stimulate rat sarcoma (RAS) and phosphoinositide 3-kinase (PI3K) signaling pathways; however, activation of the STAT5 pathway and the decreased expression of SH2 domain-containing protein tyrosine phosphatase 1 (SHP-1) are specific effects of FLT3/ITD mutations that lead to cell growth and block differentiation and apoptosis (Figure 2B) [37,38,39,40,41]. These peculiar properties of FLT3/ITD might confer a leukemogenic advantage over FLT3/TKD mutants. Results from in vitro and in vivo studies support a role of constitutively activated FLT3 in human leukemogenesis [42]. While the FLT3/ITD mutations (particularly when the allelic frequency of this mutation is high) have been associated with adverse prognosis [6], the prognostic relevance of TKD mutations is still uncertain. Data from clinical studies and meta-analysis showed that AML patients with FLT3/TKD mutations alone [43,44] or in combination with alterations in other genes resulted in a worsened outcome [45]. However, Mead et al. [46] reported a significant improvement in OS in AML patients with high levels of FLT3/TKDs. Bacher et al. [47] suggested that the prognostic effect of the FLT3/TKDs is dependent on additional mutations in different genes. The high frequency of FLT3 mutations (around 30%), the biological consequences of the constitutive activation of the FLT3 pathway in leukimogenesis as well as the adverse prognosis associated with FLT3 (particularly ITD, the most common mutation), have prompted researchers to develop and test FLT3 inhibitors [6].

Based on their specificity for FLT3, inhibitors were typically classified in first and second-generation agents. First-generation drugs are multi-targeted kinase inhibitors (midostaurin, sorafenib, lestaurtinib), showing a good in vitro inhibition of mutant FLT3. Clinically, they showed modest activity as single agents and toxicities, which is likely due to their off-target effects [48]. In a phase II study, midostaurin at a dose of 75 mg three times daily did not have sufficient clinical activity in 20 patients with relapsed or refractory FLT3-mutated AML; in addition, two patients died for treatment-related pulmonary events [49].

Nevertheless, preclinical studies evidencing an in vitro synergy between first-generation inhibitors and chemotherapy in suppressing FLT3 mutant AML cells [50,51] supported further clinical trials. A combination of sorafenib with chemotherapy in induction and postremission phases produced mixed results, and was associated with increased toxicities [52,53,54]. Lestaurtinib, a staurosporine analog with a broad spectrum of activity against tyrosine kinases, including FLT3, failed to increase response rate and prolong OS when administrated after chemotherapy in AML patients in first relapse [55]. In this randomized study (Cephalon 204 study), subjects with FLT3 mutant AML received chemotherapy alone (*n* = 106) or chemotherapy followed by lestaurtinib (*n* = 111), achieving a CR + CR with incomplete platelet recovery (CRp) in 21% and 26% of cases, respectively [55]. Interestingly, the pharmacodynamic analysis revealed that only 58% of AML patients treated with lestaurtinib reached a sufficient FLT3 inhibition (less than 15% of its baseline activity) by day 15 of therapy, and the level of inhibition correlates with the remission rate [55]. When added to first-line chemotherapy, lestaurtinib did not improve five-year OS and five-year relapse-free survival (RFS) in AML patients with confirmed FLT3-activating mutations, as shown by the meta-analyzed outcome data from United Kingdom Medical Research Council 15 (UK AML15) and 17 (UK AML17) trials [56]. As in the Cephalon 204 study, patients who received lestaurtinib and achieved a sustained FLT3 inhibition showed a reduced rate of relapse and an improved OS compared to those who did not reach a FLT3 plasma inhibitory activity (PIA) >85% [56]. Midostaurin is a pan-kinase inhibitor that was originally developed to inhibit protein C kinase, and later showed activity against both FLT3 mutations [57]. In a phase Ib trial, midostaurin was tested at the dosage of 50 mg or 100 mg twice daily in association with chemotherapy in induction and postremission therapy in newly diagnosed AML patients with mutant FLT3 and wild-type FLT3 [56]. While the 100-mg dose was not tolerated due to grade 3 nausea, vomiting, and diarrhea, the dosage of 50 mg twice daily for 14 days was well tolerated, and resulted in high complete response and high probabilities of OS at one and two years [58]. The promising results obtained in early-phase clinical studies investigating the association of midostaurin plus chemotherapy led to the phase III trial. The CALGB10603/RATIFY trial was a multicenter, double-blind placebo-controlled, phase III study that enrolled around 700 adult patients (18–59 years of age) with newly diagnosed AML and a confirmed FLT3 mutation [59]. In this study, patients (*n* = 717) were randomized to receive a standard induction (daunorubicin + cytarabine) and consolidation (high-dose cytarabine) chemotherapy plus placebo (*n* = 357) or midostaurin (*n* = 360) at a dosage of 50 mg twice daily on days 8 (from the start of chemotherapy) through 21; after completion of consolidation therapy, patients who remained in remission received midostaurin (50 mg twice daily for 12 months) or placebo. The treatment with midostaurin prolonged the event-free survival (EFS, 8.2 months versus 3.0 months; *p* = 0.002), the disease-free survival (DFS, 26.7 months versus 15.5 months; *p* = 0.01) and the OS (74.7 months versus 25.6 months; *p* = 0.009). In addition, there was a 22% reduced risk of death (HR = 0.78, *p* = 0.009) and a 21.6% lower risk of relapse (HR = 0.78, *p* = 0.002) in the midostaurin group than in the placebo group. An analysis of subgroups according to FLT3 mutation (TKD or ITD with either high or low allelic ratio) revealed that all of the patients benefited from midostaurin treatment in term of EFS and OS [59]. The rate of CR, which was defined as CR reported within 60 days of protocol therapy initiation, was 58.9% in the midostaurin arm and 53.5% in the placebo arm (*p* = 0.15); however, using an expanded definition of CR, including all of the CRs during treatment and within 30 days of treatment discontinuation, the CR rate was significantly higher in the midostaurin group (68% versus 61%; *p* = 0.04) [59]. Interestingly, 28.1% of AML patients treated with midostaurin were able to undergo allogenic HSCT during their first complete remission, in comparison with 22.7% of patients in the placebo arm (*p* = 0.10). Collectively, the rates of adverse events of grade ≥3 were similar between treatment arms, except for anemia and rash, which were more common in the midostaurin group [59]. For the first time, a phase III trial showed that the administration of a targeted therapy combined with standard chemotherapy significantly improved survival in AML. Therefore, based on the clinical data of CALGB10603/RATIFY trial, the FDA and EMA approved midostaurin for the treatment of newly diagnosed, FLT3-mutated AML patients. 

Ongoing phase II studies are currently investigating the effects of midostaurin in combination with chemotherapy specifically in patients with FLT3/ITD mutation (NCT01477606) and in combination with decitabine in elderly AML patients (NCT02634827; NCT01846624).

Quizartinib, crenolanib, and gilteritinib are second-generation inhibitors of FLT3, with higher potency and more selective inhibitory activity. Quizartinib was specifically developed to block the activity of FLT3. After a phase I study defining the maximum tolerated dose (200 mg/day) and showing an encouraging high response rates in patients with relapsed or refractory AML [60], several phase II studies have been conducted in relapsed and refractory settings using quizartinib as a single agent [61,62,63]. The primary outcome of an open-label, randomized, phase III trial on quizartinib versus salvage chemotherapy in patients with relapsed or refractory, FLT3/ITD positive AML are expected to be collected by 2018 (NCT02039726). Early-phase clinical studies also investigated the effect of quizartinib in association with chemotherapy with encouraging results [64]; therefore, a phase III trial that compared the efficacy of quizartinib versus placebo, both administered with standard induction and consolidation chemotherapy, in AML patients with FLT3/ITD mutations, is still recruiting participants (NCT02668653). However, the potential use of quizartinib in patients with FLT3/ITD mutations is limited by the resistance to quizartinib treatment due to acquired FLT3/TKD mutations [65].

Crenolanib was originally developed as a selective inhibitor of the platelet-derived growth factor receptors (PDGFR), but was found to also be a potent inhibitor of mutated FLT3, particularly the secondary mutation D835 [66]. The acquisition of a secondary point mutations in the FLT3 kinase domain (usually at residue D835 or residue F691), which may affect the FLT3 structure and function, is the underlying mechanism of resistance to FLT3 inhibitors [67]. A phase II study of relapsed/refractory AML patients with activating FLT3 mutations revealed that crenolanib (dosage of 200 mg, three times/day continuously in 28-day cycles) induced a CR with an incomplete count recovery (CRi) in 23% of FLT3/tyrosine kinase inhibitor (TKI) naïve patients, and in 5% of patients previously treated with TKI against FLT3 [68]. The addition of crenolanib (dosage of 100 mg, three times/day) to standard “7 + 3” induction chemotherapy produced the following results in patients with newly diagnosed AML patients bearing a FLT3 mutation: overall, 96% of patients achieved a CR with full count recovery, while 88% achieved CR after one cycle of induction; with a median follow up of six months, only three patients aged >60 years relapsed [69]. With these encouraging results, a phase III study was designed to investigate the efficacy of crenolanib versus midostaurin in AML patients with FLT3 mutations; both targeted therapies will be administered following induction chemotherapy, consolidation chemotherapy, and bone marrow transplantation (NCT03258931).

In vitro and in vivo studies demonstrated that gilteritinib, a pyrazinecarboxamide derivative also known as ASP-2215, is a selective and potent inhibitor of FLT3 activating mutations [70]. In addition, this anti-FLT3 agent showed activity against AXL, which is a member of the TAM (TYRO 3, AXL, and MER) receptor tyrosine kinase subfamily that seems to play as a modulator of FLT3 activity [70]. The CHRYSALIS trial was an open-label phase I/II study that assessed the safety and tolerability, pharmacokinetic and pharmacodynamic profiles, and antileukemic activity of gilteritinib in a large cohort of relapsed/refractory AML patients (*n* = 252) [71]. In this first-in-human study, gilteritinib was generally well tolerated, with a maximum tolerated dose of 300 mg, and clinically active at the dose of ≥80 mg once daily, in patients with FLT3 mutations [71]. Therefore, several phase III studies have been planned to test gilteritinib versus salvage chemotherapies in AML patients with FLT3 mutations in a relapsed/refractory setting (NCT02421939, NCT03182244). A phase I study is recruiting now newly diagnosed AML patients primarily to define the safety and tolerability profile of gilteritinib administered in combination with induction and consolidation chemotherapy (NCT02236013).

## 5. IDH1/2 Inhibitors

Isocitrate dehydrogenase 1 and 2 (IDH1 and IDH2) are metabolic enzymes located in the cytoplasm/peroxysomes and mitochondria, respectively, that catalyze the oxidative decarboxylation of isocitrate to α-ketoglutarate. Point mutations in these proteins have been found to be associated with several malignancies, including AML. In particular, IDH1 and IDH2 mutations account for 20% of all AML cases, affecting 7–14% and 8–19% of patients, respectively [72]. In vitro and in vivo studies proved that mutations in IDH proteins decrease their enzymatic activity as well as confer a gain of function activity to convert α-ketoglutarate to d-2-hydroxyglutarate (D-2HG) [73]. D-2HG and α-KG share similar structures; therefore, D-2HG can act as a competitive inhibitor of the other metabolite, interfering with α-KG activities such as cellular metabolism and epigenetic regulation [73]. The dysregulation induced by α-KG translates in an arrest of cell differentiation, and thus oncogenesis. Novel therapeutic approaches have been developed to target leukemic cells carrying IDH1/2 mutations (AG-120, AG-221, BAY1436032, IDH305, AG-881, FT-2102) [74]. 

The specific inhibitor of IDH1, ivosidenib (AG-120), was evaluated for the first time in a clinical trial as a single agent in patients with IDH mutation-positive advanced hematologic malignancies [75,76,77]. Doses ranged from 300 mg to 1200 mg once daily, with a subgroup of patients that received the dosage of 100 mg twice per day. All ivosidenib doses tested in the pharmacokinetic/pharmacodynamic analysis were able to reduce the D-2HG plasma level [76]. The maximum tolerated dose was not defined, and the most common grade ≥3 AEs (≥15%) were febrile neutropenia, anemia, leukocytosis, and pneumonia [77]. Among 78 patients with evaluable response, 30 patients experienced an ORR (38.5%) and 14 patients (17.9%) experienced a CR [75,77]. A variant allele frequency (VAF) analysis using next-generation sequencing (NGS) demonstrated that ivosidenib treatment resulted in a clearance of mutant IDH1 in 27.3% of patients with CR [77]. In a recent phase I study, CR, CR + CRi, and ORR were observed, respectively, in 21.6%, 30.4%, and 41.6% of patients with IDH1-mutated AML receiving ivosidenib 500-mg monotherapy, with a median duration of 8.2 months, 9.3 months, and 6.5 months [78]. 

Enasidenib (AG-221) is an oral, selective inhibitor of mutant IDH2 that presented an acceptable tolerability profile, with 41% of 239 AML patients (113 in the dose-escalation phase and 126 in the four-arm expansion phase) reporting grade 3–4 treatment-emergent adverse events (hyperbilirubinemia, 12%; IDH differentiation syndrome, 6%; thrombocytopenia, 6%), in a first-in-human, phase 1/2 study [79]. Besides the safety and tolerability, this trial also evaluated the pharmacokinetic and pharmacodynamic characteristics of enasidenib in an expansion phase (dose of 100 mg once daily), showing clinical efficacy [79]. Among 176 patients (74%) with relapsed/refractory disease and IDH2 mutation, 40.3% achieved an ORR and 19.3% achieved CR. The median OS was 9.3 months in all of the relapsed/refractory patients, and 19.7 months in those patients with relapsed/refractory disease who reached a CR [79]. Based on these promising data, enasidenib was recently approved by the FDA in advanced mutant IDH2 AML. The multicenter, open-label, randomized, phase III study IDHENTIFY is currently recruiting elderly subjects (≥60 years) with IDH2 mutant AML to compare enasidenib treatment to conventional care regimens (NCT02577406).

The safety profiles of AG-120 and AG-221 in combination with standard induction and consolidation therapy are being currently investigated in a phase I study in patients with IDH-mutant AML (NCT02632708).

## 6. BCL-2 Antagonists

Apoptosis is a specific morphological aspect of cell death that is characterized by the elimination of cells through the activation of two distinct pathways: the intrinsic pathway is mainly triggered by apoptogenic factors released from mitochondria, while the extrinsic pathway is initiated by extracellular signals [80]. The family of the B cell lymphoma 2 (BCL-2) protein regulates the intrinsic pathway through the interaction between antiapoptotic factors [BCL-2, BCL-XL, BCL-W, myeloid cell leukemia sequence 1 (MCL1), BFL-1/A-1], and proapoptotic factors [BCL-2 homology 3 (BH3) domain-only proteins] [81]. Alterations in the apoptotic mechanisms are key elements for tumor progression. In particular, the overexpression of prosurvival BCL-2 family members has been found to play a crucial role in the development and progression of AML [82,83,84]. Venetoclax (ABT-199) is a mimetic of the BH3 domain that specifically antagonizes BCL-2 activity. In vitro and ex vivo preclinical studies demonstrated that AML cells are very sensitive to treatment with this pharmacologic agent [85]. The favorable safety/tolerability profile and the encouraging clinical outcomes of venetoclax treatment in association with low dose chemotherapy [86] or HMAs [87] in elderly (≥65 years) subjects with treatment-naive AML who are not eligible for an intensive anthracycline-containing induction chemotherapy, led to the design and development of randomized phase III trials (NCT03069352, NCT02993523). In this disease setting, 600 mg of venetoclax administered once daily in association with low-dose cytarabine (LDAC) led to an ORR in 75% of patients, a CR + CRi in 70% of patients, and a 12-month OS of 74.7% [86]. Among 57 patients treated with venetoclax with decitabine or azacitidine, 35 (61%) achieved a CR + CRi [87].

## 7. New Histone Deacetylase Inhibitors and Hypomethylating Agents

In the last decade, several mutations of genes involved in epigenetic mechanisms have been identified in AML, through whole genome sequencing, exome sequencing, and targeted sequencing analyses. Epigenetic modifications that have been found altered in AML include DNA methylation (DNA methyltransferase 3 alpha, DNMT3A, IDH1 and 2, ten-eleven translocation-2, TET2) and chromatin modifications (ASXL1, EZH2) [88]. Both 5-azacitidine and decitabine are DNA-hypomethylating agents that showed convincing clinical activity and tolerability as monotherapy in AML, and now represent the standard of care for older AML patients (aged ≥65 years) and those who are ineligible for more intensive therapies [89]. Epigenetic-modifying drugs should be considered mechanism-targeted rather than specific mutation-targeted agents. Novel agents are now in preclinical development or have just entered the early clinical trial phases, such as guadecitabine and pracinostat.

Guadecitabine is a dinucleotide of decitabine and deoxyguanosine that presents a prolonged half-life in comparison with first generation hypomethylating agents. A multicentre, open-labeled phase I/II study (NCT01261312) investigated the safety and activity of this drug in treatment-naïve, older AML patients [90], and in those with refractory/relapsed malignancy [91]. Many doses of guadecitabine in different schedules were evaluated: 60 mg/m^2^ or 90 mg/m^2^ on days 1–5 (five-day schedule) of a 28-day treatment cycle and 60 mg/m^2^ in a 10-day schedule of a 28-day treatment cycle. In previously treated patients, the composite CR (CRc) was achieved in 23.3% of patients, with a higher response observed in the 10-day schedule group versus the five-day schedule group [91]. In treatment-naïve patients, no statistically significant differences in term of CRc were observed between dose groups or schedules (54% with 60 mg/m^2^ on the five-day schedule, 59% with 90 mg/m^2^ on the five-day schedule, 50% with 60 mg/m^2^ on the 10-day schedule) [90]. Phase III studies investigating the efficacy of this hypomethylating agent in both disease settings are ongoing (NCT02348489, NCT02920008).

Pracinostat belongs to the group of histone deacetylase inhibitors (HDACIs); these drugs are known to exert antiproliferative and cytotoxic activities in tumor cells [92]. This orally active HDACI was evaluated in early clinical trials both as a single agent [93] and in combination with HMAs [94] in patients with hematologic malignancies. In previously untreated elderly AML patients who were unsuitable for intensive chemotherapy, the combination of pracinostat + azacitidine produced a remarkable response. Among 50 evaluable patients, 27 (54%) reached the primary endpoint of CR + CRi + morphologic leukemia free state (MLFS), including 21 patients (42%) who achieved a CR [94]. A phase III study is currently evaluating this drug’s combination versus placebo + azacitidine in newly diagnosed AML patients who are unfit to receive intensive chemotherapy due to age ≥75 years or comorbidities (NCT03151408).

## 8. Conclusions

The standard treatment in AML includes an intensive induction chemotherapy followed by a postremission therapy with HSCT or chemotherapy. However, the modern management of AML has been significantly improved by the availability of novel targeted agents as well as drugs with novel cytotoxic delivery approaches. Besides conventional treatments and new therapeutic strategies, palliative medication should also be taken into account for the optimal management of AML patients. 

The World Health Organization (WHO) defines palliative care as an approach that can improve the quality of life (QoL) of patients and their families through the timely identification of deteriorating health, holistic assessment of needs, management of pain and other symptoms (physical, psychosocial, and spiritual), and the proposal of a person-centered planning of care [95]. Randomized and observational studies have shown that patients with advanced solid tumors who were assigned to receive early palliative care (EPC) integrated with standard care, compared to patients assigned to standard oncologic care alone, reported improvements in QoL, lower depression, lower rates of intravenous chemotherapy use near death, as well as superior awareness of prognosis and longer survival [96,97]. Relevant to this, the American Society of Clinical Oncology (ASCO) and the European Society of Medical Oncology (ESMO) have recognized that patients with advanced solid cancer should receive dedicated palliative care services, concurrent with active treatment, early in their disease trajectory, preferably within eight weeks from diagnosis [98,99]. The importance of the EPC approach has only been recently recognized in the hematologic scientific and clinical community [97]. In the only one randomized study published so far, enrolling patients undergoing hemopoietic stem cell transplantation for hematologic malignancy, the use of inpatient EPC, compared with standard transplant care, resulted in a smaller decrease in QoL two weeks after transplantation [100]. After approximately 40 years of none or little advancements in the treatment of this malignancy, new therapeutic options such as targeted therapies (midostaurin and enasidenib), monoclonal antibodies (gemtuzumab ozogamicin), and a novel liposomal formulation of cytarabine and daunorubicin (CPX-351), will be soon available for adult patients with AML. Table 1 summarizes the approved or selected emerging therapies for the treatment of AML that were previously described. 

So far, combination therapy based on traditional cytotoxics plus personalized treatments, rather than monotherapy, seem to offer stronger antileukemic effects and higher response rates, with manageable toxicities. Elderly, refractory, or relapsed patients and selected patients with AML-related genetic factors particularly benefit from specific targeted therapies in combination with standard regimens. Reducing side effects while maintaining or increasing efficacy represents the new challenge in AML treatment. This aim could be achieved, for instance, by combining novel agents and reducing the use of high-dose chemotherapy. Future research studies should also focus on AML patients in order to define their early palliative care needs and investigate whether the anticipated offer of palliative and supportive care to AML patients receiving either standard or innovative therapies might positively impact on their QoL, and clinical outcome.

## Figures and Tables

**Table 1:** Approved or selected emerging therapies for the treatment of AML.

Agent	Mechanism of Action	Characteristics of Patients	Monotherapy/Combination Therapy	Latest Phase of Development */Approved Agent	References
**Monoclonal antibodies**					
Vadastuximab talirine	Anti-CD33 antibody conjugate	Patients with CD33-positive AML	Single agent/with standard chemotherapy/with HMAs	1	[11,12,13,14]
Gemtuzumab ozogamicin	Anti-CD33 antibody conjugate	Patients with newly diagnosed, CD33-positive AML	With standard chemotherapy	Approved by FDA and EMA	[18,19,20]
Nivolumab	Anti-PD-1	Adult patients with relapsed or refractory AML	With azacitidine	1/2	[21]
**New formulation of old drugs**					
CPX-351	Liposomal formulation of cytarabine and daunorubicin	Adult patients with newly diagnosed therapy-related AML (t-AML) or AML with myelodysplasia-related changes	Single agent	Approved by FDA	[29,30,31,32]
**FLT3 inhibitors**					
Sorafenib	Multi-targeted kinase inhibitor	Adult or older patients with AML/patients with FLT3 mutant AML	With standard chemotherapy/with azacitidine	2	[52,53,54,101,102]
Lestaurtinib	Multi-targeted kinase inhibitor	Adult patients with FLT3 mutant AML in first relapse/adult patients with newly diagnosed, FLT3 mutant AML	With salvage chemotherapy/with standard chemotherapy	2	[55,56]
Midostaurin	Multi-targeted kinase inhibitor	Adult patients with newly diagnosed FLT3 mutant AML	With standard chemotherapy	Approved by FDA and EMA	[58,59]
Quizartinib	Second generation FLT3 inhibitor	Adult patients with relapsed or refractory AML	Single agent	2	[61,62,63]
Crenolanib	Second generation FLT3 inhibitor	Adult patients with relapsed or refractory, FLT3 mutant AML/adult patients with newly diagnosed, FLT3 mutant AML	Single agent/with standard chemotherapy	2	[68,69]
Gilteritinib	Second generation FLT3 inhibitor	Adult patients with relapsed or refractory AML	Single agent	1/2	[71]
**IDH1/2 inhibitors**					
Ivosidenib	IDH1 inhibitor	Adult patients with relapsed or refractory, IDH1 mutant AML	Single agent	1	[75,76,77,78]
Enasidenib	IDH2 inhibitor	Adult patients with relapsed or refractory AML, IDH2 mutant AML	Single agent	Approved by FDA	[79]
**BCL-2 antagonists**					
Venetoclax	BCL-2 inhibitor	Older patients with untreated AML, ineligible for standard induction therapy	With low-dose cytarabine/with HMAs	1/2	[86,87]
**New histone deacetylase inhibitors and hypomethylating agents**					
Guadecitabine	DNA methyltransferase inhibitor	Older patients with untreated AML/adult patients with relapsed or refractory AML	Single agent	2	[90,91]
Pracinostat	Histone deacetylase inhibitor	Older patients with untreated AML, ineligible for intensive therapy	With azacitidine	2	[94]

* The latest phase of clinical studies published (a systematic query-based MEDLINE search was carried out using the following keywords: “name of drug”, “clinical study”, “acute myeloid leukemia”) or presented at International meetings. AML: acute myeloid leukemia; HMAs: hypomethylating agents; FDA: Food and Drug Administration; EMA: European Medicines Agency.
